# Intravenous Thrombolysis After Reversal of Dabigatran With Idarucizumab in Acute Ischemic Stroke: A Case Report

**DOI:** 10.3389/fnagi.2021.765037

**Published:** 2021-12-14

**Authors:** Dan Xie, Xuefan Wang, Yao Li, Ruiling Chen, Yingying Zhao, Chunling Xu, Qian Zhang, Yongbo Zhang

**Affiliations:** ^1^Department of Neurology, Beijing Friendship Hospital, Capital Medical University, Beijing, China; ^2^Department of Neurology, Beijing Tiantan Hospital, Capital Medical University, Beijing, China

**Keywords:** acute ischemic stroke, dabigatran, anticoagulation, intravenous thrombolysis, idarucizumab

## Abstract

**Background:** As there is a growing concern about the cerebral embolism events secondary to non-valvular atrial fibrillation (NVAF), novel oral anticoagulant (NOAC) has been more and more widely used as an anticoagulation treatment for the prevention of stroke. However, in the face of life-threatening bleeding or emergency surgery/treatment, NOAC-related antagonists such as idarucizumab need to be urgently used to reverse the NOAC. Using recombinant tissue plasminogen activator (rt-PA) intravenous thrombolysis for acute ischemic stroke requires a time window of 4.5 h. This case reports rt-PA intravenous thrombolysis after reversal of dabigatran anticoagulation with idarucizumab in patients with acute ischemic stroke.

**Case Presentation:** We report the case of 62-year-old Chinese female with NVAF treated with dabigatran 110 mg twice daily, and missed a dose on the eve of the stroke. The patient presented with acute ischemic stroke causing the angle of mouth deviated to right side and left limb weakness in the early morning of the next day. However, the last dosing time of dabigatran was between 24 and 48 h, the patients were given rt-PA intravenous thrombolysis after reversal of dabigatran anticoagulation with idarucizumab, while any potential relative contraindication had been excluded by means of laboratory test and CT scan in the hospitalization services. National Institute of Health stroke scale (NIHSS) score was reduced from 4 to 1, and the patient was discharged after 2 weeks.

**Conclusion:** Our case report adds to the evidence that idarucizumab administration is safe and effective in the setting of patients with atrial fibrillation treated with dabigatran who develop acute ischemic stroke requiring rt-PA intravenous thrombolysis.

## Background

Acute ischemic stroke is a serious threat to the health of Chinese residents and increased yearly. It is the leading cause of death for urban residents in China and characterized by high morbidity and lethality, high rate of recurrence and disability, and high treatment cost. Cardiac embolism secondary to non-valvular atrial fibrillation (NVAF) accounts for 13–26% of ischemic stroke patients for whom long-term and stable anticoagulant therapy is very important (Ist-3 collaborative group, 2015; Seiffge et al., 2019). Compared with warfarin, novel oral anticoagulant (NOAC) significantly reduced the incidence of stroke by 19%, among which dabigatran significantly reduced the incidence of stroke by 34%. There was no significant difference in the overall incidence of major bleeding between the two (Ruff et al., 2014). Thus, clinical application of NOAC has been both recommend by “2019 AHA/ACC/HRS Guideline for the Management of Patients with Atrial Fibrillation” and the “Guideline of stroke prevention in Chinese patients with atrial fibrillation (2017)” (Zhang et al., 2017; January et al., 2019). In order to save the patients’ lives and improve the prognosis, it is particularly critical to treat the patients with corresponding antagonists when life-threatening bleeding or emergency surgery/treatment occurs in the users of NOAC as their widespread clinical application in China. Idarucizumab, an antagonist of dabigatran, was approved in China in February 2019. However, there is still very limited data on the efficacy and safety on idarucizumab’s use in China. Here, we report the first case of acute stroke using the recombinant tissue plasminogen activator (rt-PA) intravenous thrombolysis after the reversal of dabigatran anticoagulation with idarucizumab within the time window of thrombolysis in China.

## Case Review

The 62-year-old female patient was admitted to a neurology emergency at 8:50 a.m. on November 1, 2019, due to “sudden verbal slant accompanied by left limb weakness for 1.5 h.” She was suddenly appeared the left side of the mouth aslant, conscious of numbness and weakness of the limb on the same side, when she was having breakfast in the morning (7:20 a.m.), and could still walk by herself without headache, dizziness, unclear vision, speech deficit, or any other manifestations. Her family drove her to the hospital immediately. She had a history of hypertension for more than 30 years without regularly monitoring. The highest blood pressure was 150/100 mmHg, taking valsartan 80 mg Qd. She also had a hyperlipidemia history for 6 years, with long-term administration rosuvastatin 5 mg Qn. She was hospitalized in our Department of Cardiology 6 months ago due to “atrial flutter” and received metoprolol 12.5 mg twice daily. One month ago, she had been hospitalized again in another hospital and diagnosed as “pathological sinus syndrome and paroxysmal atrial fibrillation” due to “atrial fibrillation,” and was treated with dabigatran 110 mg twice daily but without a good compliance. She had no special family history or history of migraine. Physical examination: BP: 131/81 mmHg (right) and 130/84 mmHg (left). There was no uplift or depression in the anterior cardiac area, and the strongest apex pulsation point was 0.5–1.0 cm within the midline of the fifth intercostal left clavicle. There was no lifting pulsation, no tremor or pericardial friction. The relative voiced boundary of the heart is normal. Heart rate: 76 beats/min, regular rhythm, normal cardiac sounds, A2 > P2, no extracardiac sounds, no cardiac murmurs, and no pericardial fricative sounds in the auscultation area of each valve.

The patient had clear mind and fluent speech, equal circle of bilateral pupils, direct/indirect response to light exists, and the eyeball moves fully in each direction, without diplopia and nystagmus. The left frontal line, nasolabial groove shallow, show the mouth angle to the right, extending tongue in the center. Double soft palate lift normal, uvula in the middle, pharyngeal reflex normal. Muscle strength of left limb grade V, bilateral finger nose test and heel-knee-tibia test accurate, bilateral Babinski’s sigh negative. There were hypoesthesia of the left limb, negative response upon meningeal stimulation, and a normal water swallow test result. In the carotid auscultation area, bilateral carotid artery vascular murmur was not heard. The calculated National Institute of Health stroke scale (NIHSS) score was a total of 4 points (2 points for facial paralysis, left weakness 1 point and left numbness 1 point).

**FIGURE 1:**
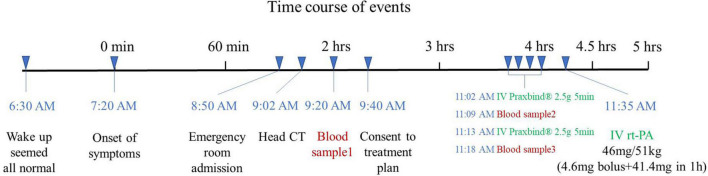
Time course of events.

The patient mentioned that she did not usually take any medication in the morning but she missed one dose of dabigatran (110 mg) the last evening after she took dabigatran at around 8 a.m. the same day. Accordingly, the last NOAC dose taking was between 24 and 48 h. Upon her emergency fast track for stroke, completed head CT, ECG, blood routine, biochemical, and coagulation function test were followed. Her head CT was normal, blood glucose was 5.5 mmol/L, and the ECG showed sinus rhythm of 72 beats/min. Coagulation function results showed activated partial thromboplastin time (APTT) 32.7 s, prothrombin time (PT) 12.3 s, International standard ratio (INR) 1.06, and creatinine clearance (Ccr) 68.62 ml/min. As ecarin coagulation time (ECT) and direct prothrombin activity were tested, idarucizumab was given at 11 a.m. with a dose of 2.5 g, followed by another 2.5 g at an interval of 5 min, and coagulation function was tested again after the administration (Table 1). The rt-PA thrombolysis therapy was initiated at 11:35 a.m. after the completion of the administration of idarucizumab, with a dose of 0.9 mg/kg. During the thrombolysis process, vital signs such as heart rate and blood pressure were monitored, and the NIHSS score was evaluated every 15 min (Figure 1). The symptoms of facial paralysis and limb numbness and weakness of the patient were significantly improved after thrombolysis. The NIHSS score was 1 (facial paralysis 1), and the patient was admitted to the stroke ward for further treatment.

**TABLE 1:** Hemostasis testing before (1), in course of (2), and after (3, 4) idarucizumab administration.

	Blood sample 1 9:20 a.m. 1/11	Blood sample 2 11:09 a.m. 1/11	Blood sample 3 11:18 a.m. 1/11	Blood sample 4 11:20 a.m. 2/11	Reference value of units
Activated partial thromboplastin time (APTT)	32.7	29.4	30.2	31.90	28.00–42.50 s
Prothrombin time (PT)	12.3	12.2	11.7	12.0	11.00–15.00 s
Normalized ratio (INR)	1.06	1.05	1.01	1.04	0.80–1.20
Fibrinogen degradation product (FDP)	0.60	0.80	2.90	1.60	0.00–5.00 μg/ml
D-dimers	0.40	0.33	0.70	0.40	0.000–1.000 μg/ml
Fibrinogen (Fbg)	2.64	2.50	2.20	2.08	2.00–4.00 g/L
Prothrombin activity [PT(A)]	88.1	89.2	94.2	91.4	70.00–120.00%
Antithrombin III	98.9	76.1	90.8	78.8	70.00–120.00%

**FIGURE 2:**
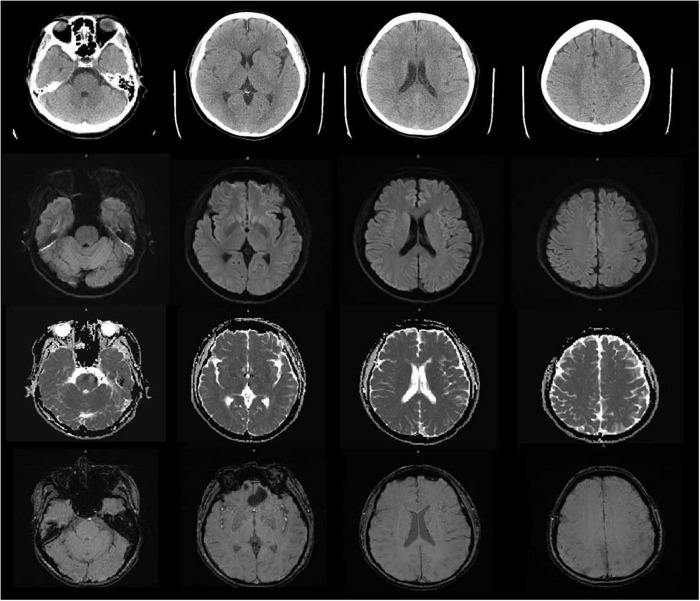
The cranial MRI showed no abnormal signal was found in T1-weighted imaging, T1WI (TI), T2-weighted imaging, T2WI (T2), diffusion-weighted imaging (DWI) and susceptibility weighted imaging (SWI).

**FIGURE 3:**
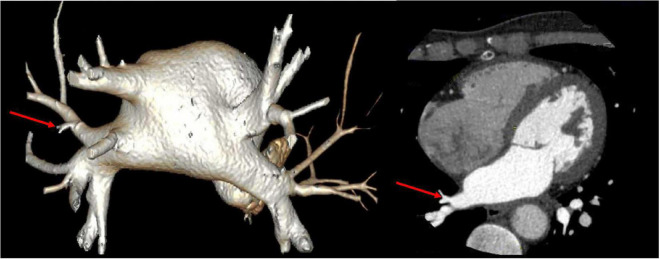
CT angiography (CTA) of left atria-pulmonary vein showed the middle branch of the right pulmonary vein was variable.

Vital signs were monitored within 24 h after admission; the patient got external auditory canal bleeding 3 h after thrombolysis. The further investigation and inquiry on her medical history showed a history of ear trauma 1 week before. Bleeding was stopped *via* external auditory canal tamponage. The cranial MRI and systemic vascular examination were arranged to find the cause of stroke, no abnormal signal was found in T1-weighted imaging, T1WI (T1), T2-weighted imaging, T2WI (T2), and diffusion-weighted imaging (DWI), and there was no microhemorrhage-related signal in susceptibility weighted imaging (SWI) (Figure 2). In the magnetic resonance angiography (MRA) measurement, intracranial vessels were indicated normal and bilateral embryonic posterior cerebral arteries were observed. Carotid ultrasound showed bilateral carotid intima thickening with plaque formation and plaque was found in the right subclavian artery. Arteriovenous ultrasound of lower extremity showed normal. Transcranial Doppler showed a rapid increase in the flow rate of the left middle cerebral artery, and 5–6 microembolic could be found in foaming experiment, supporting a direct pathway from pulmonary circulation to the systemic circulation (RLS). Cardiac systemic examination was completed: 24-h Holter showed sinus bradycardia, short atrial tachycardia, and atrial premature beats (some of which were not transmitted). Widening ascending aorta was shown on echocardiography. Thrombus was not found in bilateral atrium and left auricle in transesophageal echocardiography, also no septal shunt beam was observed in the atrial septal fossa ovale. From the data that were collected from the other hospital, left atrium was slightly larger and the middle branch of the right pulmonary vein was mutated on the CT angiography (CTA) of the left atria and pulmonary veins on 26th September (Figure 3). Diagnosis of transient ischemic attack, cardiogenic cerebral embolism, paroxysmal atrial fibrillation, pulmonary arteriovenous fistula, hypertension with Grade 2, and hyperlipidemia were observed after admission. She was suggested to restart the anticoagulant treatment deal to the CHA_2_DS_2_-VAS score was 4 and HAS-BLED score was 2. The pulmonary artery digital subtraction angiography (DSA) was also suggested after patients were discharged.

## Discussion

**FIGURE 4:**
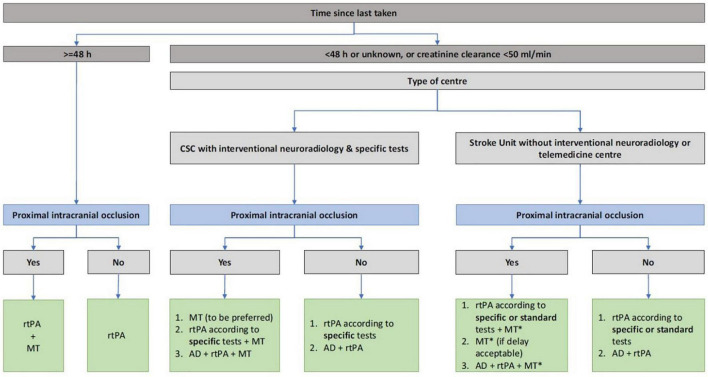
The different treatment of acute ischemic stroke in patients according to the last dosing time of novel oral anticoagulant, renal function, stroke center qualification.

In China, the anticoagulation compliance rate (INR 2.0–3.0) among the atrial fibrillation patients who are taking the warfarin was only 36% and most of their INR ware remained at <2.0 (Zhao and Huang, 2018). In recent years, the anticoagulant treatment has been effectively simplified with the marketing of direct oral anticoagulants. NOAC has been selected by more and more patients with atrial fibrillation in clinical since its efficacy and safety have been confirmed in multiple international clinical trials such as RE-LY (Poller et al., 2009; Davis et al., 2017). In our case, an embolic stroke still occurred while the patient took dabigatran 110 mg twice daily. There were two causes to be considered: (1) The dose of dabigatran was insufficient, it should be adjusted to 150 mg twice daily after stroke. (2) Although the patient was prescribed with dabigatran, she failed to follow medical advice and took the medicine regularly, which may be the incentive cause for an embolic stroke. Therefore, the dosage of dabigatran should be adjusted according to the happening of the embolization stroke and the compliance of patients should be considered in the selection of anticoagulation. In the process of NOAC, doctors and patients are also concerned about the possible life-threatening bleeding such as acute intracranial hemorrhage and massive gastrointestinal bleeding, or the emergency cases such as acute abdominal disease and fracture requiring **emergency surgery/treatment**. As a specific antagonist of dabigatran, idarucizumab, which has just been approved in China in February 2019, can bind to dabigatran irreversibly and effectively as it has an affinity for dabigatran 350 times than dabigatran for thrombin, was recommended by the European Heart Rhythm Association for the treatment of patients with bleeding and emergency surgery under oral NOAC (Steffel et al., 2018). As an exogenous humanized monoclonal antigen–antibody binding fragment (Fab), idarucizumab can quickly intravenous administration and irreversibly bind to dabigatran immediately. Idarucizumab can specifically bind to free dabigatran, dabigatran that has been bound to thrombin, and the active metabolites of dabigatran to form a complex, resulting in the inability of dabigatran to bind to thrombin, reversal the anticoagulant effect of dabigatran. Moreover, idarucizumab has no endogenous target, and no reversal effect on heparin or other anticoagulant drugs, so it does not interfere with the coagulation cascade and has no procoagulant effect (Schiele et al., 2013; Eikelboom et al., 2015; Frontera et al., 2016). In our case, intravenous infusion of idarucizumab lasted for 16 min, and rt-PA treatment could be initiated immediately 10–15 min later. The European Stroke Organization guidelines provide evidence-based recommendations to assist physicians in their clinical decisions with regard to intravenous thrombolysis for acute ischemic stroke, and recommend intravenous thrombolysis with alteplase to improve functional outcome in patients with acute ischemic stroke within 4.5 h after symptom onset (Berge et al., 2021). Idarucizumab could affect immediately that is more suitable for stroke patients within 4.5 h for intravenous thrombolysis or within 6 h for arterial thrombectomy in emergency. However, this case also has the following limitations: (1) the patient failed to initiate thrombolytic therapy within 3 h after the onset as it took a certain amount of time for her to obtain idarucizumab due to the unavailability at emergency department. (2) The time of last dose of dabigatran was between 24 and 48 h. Although coagulation indexes, such as APTT and PT and INR index, had been monitored before and after use, no obvious changes were found. The more important coagulation indexes like ECT, TT, and direct prothrombin activity were unavailable in emergency laboratory. At that point, it was difficult to decide whether rt-PA could be used without reversal of idarucizumab. It shouldbe expected to get improving in similar cases. With the application of idarucizumab, we also needed to be alerted to the occurrence of adverse events. The REVERSE-AD study showed that 0.6% of patients who used idarucizumab had hypersensitivity reactions within 5 days of administration, such as fever and bronchospasm, hyperventilation, skin rash, and itching. And other adverse reactions such as hereditary fructose intolerance, transient proteinuria also had been reported (U.S. Food and Drug Adminstration, 2015). In a French retrospective research, patients with an acute ischemic stroke treated with dabigatran within 48 h were given intravenous thrombolysis after the reversal of idarucizumab without direct prothrombin activity monitoring (Touzé et al., 2018). According to the relationship between the onset time of stroke and her last dose for dabigatran, a different treatment plan was thereby recommended, combining with different medical conditions (Figure 4). Caponi reviewed 55 thrombolytic cases after idarucizumab reversal of dabigatran showed that 81.9% (45 cases) of patients had improved NIHSS score (median 5 points), together with follow-up mRS <2 in 56% of patients, suggest that effectiveness for intravenous thrombolysis (IVT) has been preserved. In a retrospective study of 120 patients with acute ischemic/hemorrhagic stroke in Germany, the efficacy and safety of idarucizumab reversal of dabigatran in intravenous thrombolysis with an acute ischemic stroke had been confirmed (Caponi et al., 2018). There is a new data analysis which has performed a comprehensive review of 251 cases of patients with acute ischemic stroke (AIS) performed IVT after idarucizumab reversal. Regardless of stroke severity and age, there was a significant NIHSS reduction of 6 points post-stroke and linear regression revealed a correlation of admission NIHSS to NIHSS reduction (*p* < 0.001). Reassuring evidence about the safety and efficacy of this therapeutic strategy was provided (Frol et al., 2021). However, in patients with hemorrhagic stroke, the application of idarucizumab can prevent the expansion of hematoma and improve the prognosis of patients as the bleeding may be caused by dabigatran (Kermer et al., 2020). These suggest the safety and usability of reversal of dabigatran in IVT.

## Conclusion

As the first case of ischemic stroke treated with intravenous thrombolysis after idarucizumab reversal of dabigatran in China, this case was considered a mild stroke (NIHSS ≤5 points). After treatment, the NIHSS score of patient was significantly improved indicating a good prognosis. In this case, the efficacy of idarucizumab in the treatment of acute ischemic stroke patients using dabigatran was confirmed, and no directly related side-effects was found, suggesting that idarucizumab could be used as an emergency medication for patients with an acute stroke. As the only approved NOAC antagonist in China, the efficacy and safety of idarucizumab need to be confirmed in more clinical cases. Especially in the clinical application of acute stroke patients, the stratification screening in emergency situations, selection of medication, and laboratory tests still need to be improved.

## Data Availability Statement

The original contributions presented in the study are included in the article/supplementary material, further inquiries can be directed to the corresponding author.

## Ethics Statement

Written informed consent was obtained from the individual(s) for the publication of any potentially identifiable images or data included in this article.

## Author Contributions

All authors listed have made a substantial, direct, and intellectual contribution to the work, and approved it for publication.

## Conflict of Interest

The authors declare that the research was conducted in the absence of any commercial or financial relationships that could be construed as a potential conflict of interest.

## Publisher’s Note

All claims expressed in this article are solely those of the authors and do not necessarily represent those of their affiliated organizations, or those of the publisher, the editors and the reviewers. Any product that may be evaluated in this article, or claim that may be made by its manufacturer, is not guaranteed or endorsed by the publisher.
